# MetaTransformer: deep metagenomic sequencing read classification using self-attention models

**DOI:** 10.1093/nargab/lqad082

**Published:** 2023-09-11

**Authors:** Alexander Wichmann, Etienne Buschong, André Müller, Daniel Jünger, Andreas Hildebrandt, Thomas Hankeln, Bertil Schmidt

**Affiliations:** Institute of Computer Science, Johannes Gutenberg University, Staudingerweg 9, 55128 Mainz, Rhineland-Palatinate, Germany; Institute of Computer Science, Johannes Gutenberg University, Staudingerweg 9, 55128 Mainz, Rhineland-Palatinate, Germany; Institute of Computer Science, Johannes Gutenberg University, Staudingerweg 9, 55128 Mainz, Rhineland-Palatinate, Germany; Institute of Computer Science, Johannes Gutenberg University, Staudingerweg 9, 55128 Mainz, Rhineland-Palatinate, Germany; Institute of Computer Science, Johannes Gutenberg University, Staudingerweg 9, 55128 Mainz, Rhineland-Palatinate, Germany; Institute of Organic and Molecular Evolution (iomE), Johannes Gutenberg University, J.-J. Becher-Weg 30A, 55128 Mainz, Rhineland-Palatinate, Germany; Institute of Computer Science, Johannes Gutenberg University, Staudingerweg 9, 55128 Mainz, Rhineland-Palatinate, Germany

## Abstract

Deep learning has emerged as a paradigm that revolutionizes numerous domains of scientific research. Transformers have been utilized in language modeling outperforming previous approaches. Therefore, the utilization of deep learning as a tool for analyzing the genomic sequences is promising, yielding convincing results in fields such as motif identification and variant calling. DeepMicrobes, a machine learning-based classifier, has recently been introduced for taxonomic prediction at species and genus level. However, it relies on complex models based on bidirectional long short-term memory cells resulting in slow runtimes and excessive memory requirements, hampering its effective usability. We present MetaTransformer, a self-attention-based deep learning metagenomic analysis tool. Our transformer-encoder-based models enable efficient parallelization while outperforming DeepMicrobes in terms of species and genus classification abilities. Furthermore, we investigate approaches to reduce memory consumption and boost performance using different embedding schemes. As a result, we are able to achieve 2× to 5× speedup for inference compared to DeepMicrobes while keeping a significantly smaller memory footprint. MetaTransformer can be trained in 9 hours for genus and 16 hours for species prediction. Our results demonstrate performance improvements due to self-attention models and the impact of embedding schemes in deep learning on metagenomic sequencing data.

## INTRODUCTION

About 1,000 bacterial species live in the human gut, exhibiting 150 times more genes than the human genome (1). Besides aiding the digestive system, a relation to the immune system has also been documented (1,2), suggesting imbalances in the gut microbiome can result in autoimmune and allergic diseases such as diabetes type 1, multiple sclerosis, inflammatory bowel disease (IBD) and others (2,3). Therefore, the analysis of microorganisms and their interaction with the human body is an important task in understanding and treating microbiome-associated diseases.

Recent advancements in DNA sequencing technologies have propelled new methods for microbial analysis. Of special interest are metagenomic pipelines that provide deeper insights into local communities of organisms. These methods helped to identify thousands of uncultivatable species (4–6), further expanding the knowledge of environments such as the human gut.

To identify species in a metagenomic study, sequenced DNA reads typically need to be queried against a database of annotated reference genomes. The objective is to map each read to the most likely genome of origin by finding the best matching location across all reference sequences. Substrings of length *k*—called *k-mers*—are commonly used for such tasks (7). Reference databases are constructed by indexing all reference genomes by their *k*-mers and are subsequently queried using the *k*-mers of a read to find the best matches. After candidate matches are identified, they are filtered either using a seed-and-extend-approach or a taxonomy-based voting scheme in order to determine the best matching location for each read. This approach is used by many state-of-the-art mappers such as Kraken2 (8), MetaCache (9), or CLARK (10). However, new breakthroughs of deep learning in language modeling (11) and bioinformatic (12,13) suggest the potential power of metagenomic analysis via neural networks.

Recently, deep learning methods have been explored for sequence read mapping and abundance estimation to potentially overcome the reliance on frequently changing yet incomplete reference databases as well as the high memory consumption of state-of-the-art mapping approaches. Using convolutional neural networks, GeNet (14) shows the applicability of deep learning to mapping tasks while reducing the memory footprint. However, both accuracy and execution speed are inferior compared to state-of-the-art mappers. Meta^2^ (15) further explored different embedding schemes as well as deep learning techniques such as self-attention and deep sets. As a result, Meta^2^ outperforms GeNet, but still performs worse than traditional approaches.

Liang *et al.* proposed DeepMicrobes (16)—a deep learning-based approach for metagenomic read classification. By using a multi-layer and multi-architecture model (embedding, bidirectional long short-term memory (LSTM), self-attention, as well as dense layers) DeepMicrobes is able to outperform state-of-the-art *k*-mer-based mapping approaches for species and genus identification in metagenomic gut data. However, this approach exhibits several shortcomings induced by the underlying LSTM architecture. The recurrent nature of such networks tends to be more difficult to train (17) as well as less parallelizable due to the inherently sequential nature of LSTM units.

In 2017, Vaswani *et al.* (18) proposed bi-directional transformers as a neural network architecture that avoids any recurrence or convolutional components to solve tasks on sequential data. Instead, it relies on the self-attention mechanism to correlate all pairwise input tokens for later reweighting of the input sequence. The underlying inner product structure allows for straightforward parallelization of matrix products on massively parallel hardware, further being accelerated by dedicated tensor core processing units.

Bertasius *et al.* (19) argued that the less restrictive self-attention mechanism reduces inductive bias of the trainable map compared to plain convolutions that enforce strict translation equivariance of the input by construction. Self-attention further allows for mixing contributions of input tokens for the whole sequence, hence effectively covering broader receptive fields compared to convolutional cascades with thin filter kernels (20). Other desirable properties of transformers such as faster convergence of large language models when increasing the number of trainable parameters (21,22) and their remarkable few-shot capabilities when trained in a self-supervised fashion make them a popular choice for processing sequential data. In practice, the actual maximum sequence length is limited by the quadratic complexity of the attention mechanism. Alleviating this shortcoming is still part of current research. As of now, architectures based on the original idea of the transformer achieved state-of-the-art performance in almost any natural language processing task (23–27).

Lerna (28), a transformer-based model on short- and long-read data for parameter tuning of error correction tools, generates language models of the uncorrected reads and builds a perplexity metric out of this model. Using the perplexity metric, the corrected reads can be evaluated without the need of reference genomes. Further, the input embedding is discussed, deviating from character-size embeddings, as previously used in RNN approaches, to word-size embeddings as the improved data complexity yields better results. Therefore, the authors were able to improve the error correction pipeline by 18× due to the parallelization of the attention mechanism in transformer networks, the utilization of just-in-time compilation and GPU utilization.

Given their great performance in language processing, applying transformer models on sequence based bioinformatics tasks is a logical next step. Waele *et al.* (29) used transformer models on single-cell methylation data. Here, incompleteness or low coverage impedes the adoption of current single-cell DNA methylation protocols. Therefore, a model for the understanding the biological processes to impute single-cell methylation is needed. By combining axial attention with sliding window attention, the introduced GpG transformer was able to learn the interaction between neighboring CpG sites. Furthermore, it was trained on noisy data, resulting not only in imputation but also denoising capabilities while outperforming other imputation approaches.

Another transformer based approach was used for identifying bacteriophage contigs from metagenomic protein data. Here, protein-based tokens (protein cluster with high similarity) were fed into a transformer to find bacteriophage contigs in high diversity/abundance data. Due to the adoption of transformer models and good negative samples for training, PharMer was able to outperforms other deep learning approaches and mapper on multiple datasets (30).

DNABERT used a more sophisticated approach of the standard transformer to model different tasks on DNA data. It is based on the BERT language representation model, which achieved improvements on eleven natural language processing tasks and is to this date under the top performing models with its improved versions (31,32). BERT is trained in two stages, a pre-training step where an unlabeled training set was used with different task (next sentence, masked/missing information prediction) and a fine-tune step with labeled data and the desired task. Therefore, once pre-trained multiple fine-tuning possibilities can be applied on the net (11). With BERT as a foundation, DNABERT pre-trained on *k*-mer tokens (with *k* between 3 and 6) and fine-tunes the model for different tasks like transcription binding site, proximal and core promoter regions, canonical and non-canonical splice sites prediction and several more with good predictions (13). Although, the resulting models are large and training times for pre-training as well as fine-tuning are resource intensive. Furthermore, only small *k*-mers were tested which could hamper the prediction capabilities as described by DeepMicrobes and DNABERT, showing a better performance with higher *k*-mers.

In this paper we introduce MetaTransformer, a novel self-attention-based architecture for metagenomic read classification. Our goal is to improve the classification speed as well as the prediction accuracy of deep learning approaches for classification tasks. We reduce the memory footprint, improve the computational time for both inference and training, compared to previous works, and analyze different embedding schemes. As a result, the model can even be executed on a single modern consumer-based GPU. Furthermore, we explore additional optimization strategies such as sparse embedding gradients and mixed-precision computation. We subsequently evaluate our proposed model against DeepMicrobes, as well as traditional *k*-mer-based methods.

## MATERIALS AND METHODS

All datasets are summarized in Table 1.

**Table 1.** List of all datasets

Name	Origin	Description
training	*HGR UMGS* ^[1]^	Sampled reads (coverage of 4)
validation	*HGR UMGS* ^[1]^	Sampled reads (coverage of 1)
*BenchmarkGut*	ERP108418^[2]^	10,000 sampled reads per genome
*BenchmarkMock*	ERP105624^[3]^	10 sets of 10,000,000 reads
	ERP01221^[4]^	randomly drawn
*AbsentSpecies*	RJNA348753^[5]^	Sampled reads (coverage of 1)
real-world data	PRJNA398089^[6]^	Reads with removed host DNA

An overview over all datasets used in this work, their origin and in which context the dataset is used. The original data was retrieved using the following sources:

^[1]^ http://ftp.ebi.ac.uk/pub/databases/metagenomics/umgs_analyses/

^[2]^ https://www.ebi.ac.uk/ena/browser/view/PRJEB26432

^[3]^ https://www.ebi.ac.uk/ena/browser/view/ERP108418

^[4]^ https://www.ebi.ac.uk/ena/browser/view/ERP01221

^[5]^ https://www.ebi.ac.uk/ena/browser/view/RJNA348753

^[6]^ https://www.ebi.ac.uk/ena/browser/view/PRJNA398089

### Training data

**Figure 1.**
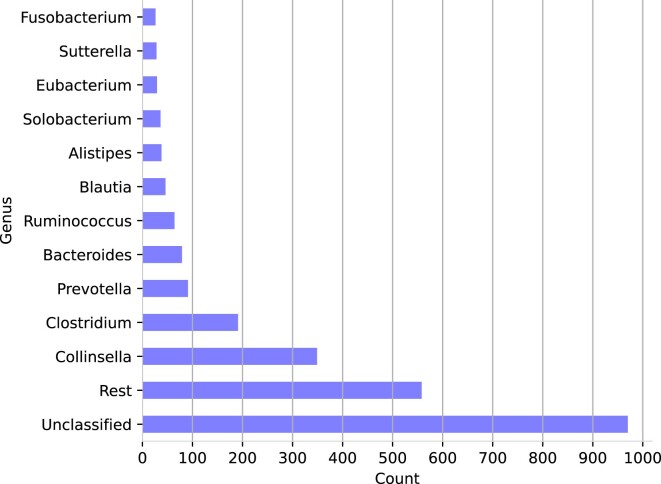
Composition of the *HGR UMGS* (4) microbial dataset. Count at genus-level for the *HGR UMGS* dataset based on 2,505 species. Only the Top 10 genera are displayed, the remaining taxa are summarized as ‘Rest’. The ‘Unclassified’ genera have a higher order classification.

For training of the species and genus classification model, sequence data provided by Almeida *et al.* (4) was used. It consists of 1,952 unclassified metagenomic species (UMGS) and 553 species from the human gut reference (HGR). The dataset will be referred to as *HGR UMGS*. UMGS data was obtained during the reconstruction of 92,143 metagenome-assembled genomes originating from 11,850 different human gut microbiomes (4). The quality of the metagenome-assembled genomes (MAGs) was assessed using CheckM (33). Only sequences exhibiting at least 50% completeness and less than 10% contamination were kept. For species-level training, all sequences spanning 2,505 classes were used. On genus level, only sequences with genus assignment were kept. This resulted in 1,535 of the 2,505 MAGs contributing to genus level training. A rough overview of the distribution of the ten most frequent genera can be found in Figure 1.

#### In-silico sequence read generation

All models were trained and evaluated using short-read data. Unfortunately, large-scale real-world datasets often lack the necessary ground truth labels needed for model optimization and evaluation. We thus used artificially generated read datasets stemming from *HGR UMGS* genomes by simulating reads using the ART Illumina read simulator (34). ART generates reads based on empirically determined profiles of sequencing technologies that include read error models as well as base quality values. For training, we generated 150 bp paired-end reads with an insert size of 400bp and a standard deviation of 50 bp. The error model corresponds to the Illumina HiSeq2500 sequencing technology. Paired-end reads were treated as single-end reads during training for all models.

Prior to training, we shuffled all reads randomly. Shuffling was performed to provide a diverse set of species or genera in each training batch in order to avoid local optima convergence during the first few iterations. We used the same set of source reference genomes for the validation data, but selected a different random seed for read generation. Shuffling was also applied to the validation data. Separate training and validation datasets were created for each taxonomic level of interest, i.e. species and genus. The number of generated reads was scaled to allow the models to converge with respect to the validation data without reusing the same reads multiple times during training.

To prevent class imbalance effects, the coverage needed to be adapted to compensate for varying genome sizes. In order to obtain a balanced training dataset we used the following approach:

- Input: *N* genomes *G*_1_, ..., *G*_*N*_ and coverage factor *f*
- Determine genome lengths *L*_1_, ..., *L*_*N*_ and the maximal genome length *L*_*max*_
- Determine per-genome coverage $C_i=\lfloor \frac{L_{max}}{L_i}\rceil \cdot f$ for each *G*_*i*_
- For each *G*_*i*_ invoke ART tool with coverage *C*_*i*_

Since we have control over the number of samples generated and are not restricted by a fixed-sized dataset, we opted for dataset-level balancing. For the training sets a coverage factor of 3 and 1 for validation was used.

### Evaluation data

#### Human gut benchmark data

To evaluate the trained models, we used two benchmark datasets provided by the authors of DeepMicrobes (16). The first one contains 3,269 high-quality MAGs obtained from an assembly (accession ERP108418) (4) of which only MAGs with at least 90% completeness, 0% contamination, and 0% strain heterogeneity were kept. For labeling, the following steps were applied: First, MASH (35) was employed to identify the closest corresponding pairs between benchmark and training genomes with the lowest MASH distance. Those candidates were subsequently globally aligned using MUMer (36). Benchmark genomes which exhibit at least 60% AQ (fraction of query aligned to reference) and 95% ANI (average nucleotide identity in the aligned fractions), regarding the alignment, were labeled with the same species as the matched training genome. For the resulting 3,269 genomes, 10,000 in-silico paired-end reads per genome were generated in the same manner as the training reads. Since the training MAGs originate from the same assembly, only MAGs which are not used for training were included. For further information the script for labeling the MAGs is available at https://github.com/Finn-Lab/MGS-gut. The dataset will be referred to as *BenchmarkGut*.

#### Mock community data

We used 10 mock communities created by Liang *et al.* (16). To generate these communities, whole genome sequenced isolates from two studies (ERP105624 and ERP012217 (37)) were used of which *N* = 258 samples with genus level annotations were selected. The abundance of each member of a community was drawn from a log-normal distribution with μ = 1 and σ = 2.

Let *A*_1_, ..., *A*_*N*_ denote the observations drawn from this distribution. The mock communities contain *M* = 10,000,000 samples. Each observation *A*_*i*_ can be transformed into an actual read count *R*_*i*_ by normalizing its value by the sum of observations and subsequent scaling according to the desired read count


(1) \begin{eqnarray*} R_i = \frac{A_i}{\sum _{j=1}^{N}A_j}\cdot M. \end{eqnarray*}

A different random seed for drawing from the log-normal distribution was used for each mock community. Reads of length 100bp were generated with ART using the same parameters as for the *BenchmarkGut* dataset. The resulting dataset will be referred to as *BenchmarkMock*.

#### Species absent from reference data.

The absent species dataset created by Liang *et al.* is based on 7,903 species genomes accessible via NCBI BioProject Accession PRJNA348753 (38). These genomes were aligned against the 2,505 genomes used for training. After aligning, genomes with an ANI of < 95% and an AQ < 60% to the closest relatives as well as an overall AQ > 10% were classified as ‘absent’ resulting in 121 genomes. A new dataset was simulated from the ‘absent’ genomes using ART as described in the subsection *In-Silico Read Generation*. Like for the validation data, we used a new seed and a coverage of one. The dataset is referred to as *AbsentSpecies*.

#### Multi-omics inflammatory bowel disease database

To test the usability of our approach on real-world data we downloaded 106 microbioms from subjects with and without inflammatory bowel disease via the SRA BioProject PRJNA398089 (39). Thereby, we used the same randomly chosen samples as DeepMicrobes consisting of 26 healthy subjects, 50 subjects with Crohn’s disease (cd) and 30 subjects with ulcerative colitis (uc). All samples were preprocessed using Trimmomatic (v. 0.33) (40) with a minimum read length of 75bp and KneadData (v 0.12.0) to remove host reads. After preprocessing we calculated the abundance using MetaTransformer. To analyze differential microbial expression in the three groups we used the linear discriminant analysis effect size (LEfSe) (41). This consists of a non-parametric Kruskal-Wallis test to determine whether at least one group (uc, cd or control) is significantly (*P* < 0.05) different from another for each species and a linear discriminant analysis (LDA) model build on these significant species to assess its effect size. Species with an LDA effect size of >2.0 were considered to be significant.

### Transformer-encoder network

In the following, we introduce our encoder-only architecture. Consider an input sequence of embedded tokens $S=[x_1,..,x_n]^T\in \mathbb {R}^{n \times d_{model}}$ extracted from a sequencing read. Here, *n* denotes the number of tokens and *d* the dimension of embedding vectors. Different embedding techniques (LSH-EmbedPool (15), Hash-EmbedPool (15), *k*-mer embedding (16)), as well as different tokenizations (character-level, byte-pair (42), *k*-mer) were tested. Both tokenizations and embeddings schemes are further explained in subsection *Embedding Schemes and Tokenization* as well as Supplementary Figure S1 in the Supplement.

#### Positionial encoding

Before the embedded tokens are passed to the next layer, they are aggregated by means of positional encoding to inject the notion of position into token embeddings. Hence, the position of sequence elements can be preserved without recurrence or convolution. Positional encoding can be a scalar or a vector for each element of the sequence and fixed or learned during training.

Here, we use a fixed positional encoding based on sine and cosine functions (18). For a position *p* ∈ {1, ...*n*} the positional encoding is a vector of dimension *d*_*model*_ defined as


**Figure 2.**
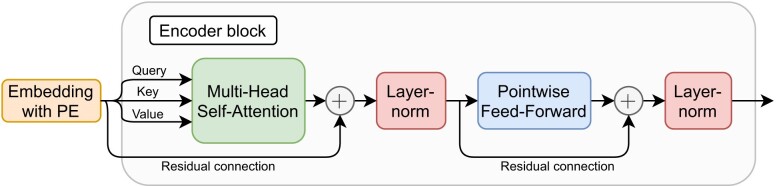
Transformer encoder block design. The encoder block consists of a multi-head self-attention module, followed by layer normalization, a point-wise feed-forward layer and another layer normalization. Before each layer normalization, a residual connection re-adds the input of the previous module.

(2) \begin{eqnarray*} &pos_{trig}(p, c) = \left\lbrace \begin{array}{@{}l@{\quad }l@{}}\sin (f_k\cdot p) & \text{if}\ j = 2k \\ \cos (f_k\cdot p) & \text{if}\ j = 2k + 1 \end{array}\right.\nonumber \\ &f_k = \frac{1}{10,000^{2k/d_{model}}} \quad \quad \quad k\in \mathbb {N} \end{eqnarray*}

where *p* denotes the current position in the sequence and *j* the *j*th component of the positional encoding vector for position *p*. The frequency of the sinusoidal functions is given by *f*_*k*_ which denotes a geometric progression from 1 to $\frac{1}{10,000}$. Therefore, the wavelengths increase monotonically from 2π to 2π · 10,000 over the dimensions of the positional encoding vector. The resulting positional encoding is robust with respect to uniqueness, it is symmetrically bounded between −1 and 1, and the network might learn to attend relative positions since it can be expressed as a linear mapping. The complete encoding for all sequence positions forms a matrix $P\in [-1, 1]^{n\times d_{model}}$. It is added point-wise to the token embedding resulting in a final input encoding $E = (S \odot \sqrt{d_{model}}) \odot P$. The result *E* is passed to the next layer which is the first of potentially multiple encoder blocks. An overview of an encoder block is shown in Figure 2.

#### Multi-head attention

The first and most important element of the encoder block is the multi-head self-attention module. We first describe the single attention-head variant before extending it to multiple heads. Given the input encoding $E\in \mathbb {R}^{n\times d_{model}}$ three different representations called query (*Q*), key (*K*) and value (*V*) are derived by means of a linear transformation of *E* given by


(3) \begin{eqnarray*} Q = EW^Q \quad K = EW^K \quad V = EW^V, \end{eqnarray*}

where $W^Q,W^K,W^V\in \mathbb {R}^{d_{model}\times d_{model}}$ are trainable parameters of the network. The self-attention mechanism is given by


(4) \begin{eqnarray*} \text{Attention}(Q, K, V) = \text{softmax} \biggl (\frac{QK^T}{\sqrt{d_{model}}}M \biggr )V \end{eqnarray*}

The dot products between the sequence elements in *Q* and *K* indicate the ‘similarity’ of the respective elements. Further, the softmax function is subsequently applied row-wise on $QK^T\in \mathbb {R}^{n\times n}$. The resulting matrix contains a set of weights in each row to compute the weighted sum of the input values $V\in \mathbb {R}^{n\times d_{model}}$. An optional mask matrix $M\in \mathbb {R}^{n\times n}$ can be used to manually restrict the network to attend specific tokens of the sequence. This could be for example positions in the sequence that denote padding. To mask the token at position *p* so that it can not be attended, the *p*-th column of *M* would be set to −∞.

The scaling factor $1/\sqrt{d_{model}}$ works analogously to standard dot product attention (43). With large values of *d*_*model*_ the dot product produces bigger results possibly leading to extreme values after the softmax function is applied. These values in turn could lead to vanishing gradients. To counteract this problem, the values of the dot product are scaled by *d*_*model*_. To allow the model to attend information based on varying patterns multiple attention-heads are employed. They are computed as


**Figure 3.**
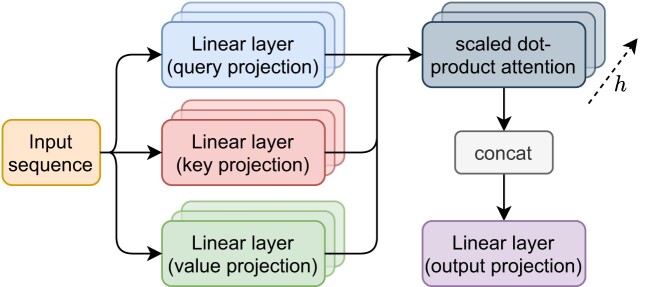
Multi-head self-attention (transformer model). An exemplary multi-head-attention module. The stacked blocks indicate the computation of the scaled dot product attention by different attention-heads in varying vector sub-spaces.

(5) \begin{eqnarray*} &Z = \text{MultiHeadAttention}(E) = \text{Concat}(a_1,...,a_h) \cdot W^O \nonumber\\ \end{eqnarray*}

(6) \begin{eqnarray*} &a_i = \text{Attention}(EW_i^Q, EW_i^K, EW_i^V) \quad \quad i \in \lbrace 1,...,h\rbrace ,\nonumber\\ \end{eqnarray*}

where *h* denotes the number of attention-heads and $W_i^Q\in \mathbb {R}^{d_{model}\times d_k}, W_i^K\in \mathbb {R}^{d_{model}\times d_k}$, $W_i^V\in \mathbb {R}^{d_{model}\times d_v}$ and $W^O\in \mathbb {R}^{(h\cdot d_v) \times d_{model}}$ are trainable parameters of the network. Here, each attention-head *i* maintains its own set of weights *W*_*i*_. Figure 3 illustrates our multi-head self-attention design. Before passing the result *Z* to the next layer, the result is concatenated and linearly transformed by *W*^*O*^ to retain an equal dimension of *d*_*model*_ throughout the whole architecture.

Adding more heads inevitably increases the computational complexity of the layer. To counteract this problem, each attention-head operates on an attention sub-space whose size inversely scales with the number of attention heads *h*. By setting *d*_*k*_ = *d*_v_ = *d*_*model*_/*h* the complexity of the layer is invariant with respect to the number of attention heads. The result *Z* is added to the original input *E* through a residual connection *Z*′ = *Z* + *E* before being passed to a layer normalization (44) over the feature dimension. Layer normalization is further explained in the subsection *Layer Normalization* in the Supplement.

The normalized elements of the sequence in *N* are subsequently passed to a (fully-connected) multilayer perceptron with a single hidden layer that is applied to each position of the sequence independently.


(7) \begin{eqnarray*} \mathrm{MLP}_i(N) = \mathrm{ReLU}(N_iW_1 + b_1)W_2 + b_2 \quad N_i\in \mathbb {R}^{d_{model}} \end{eqnarray*}

Here, $W_1\in \mathbb {R}^{d_{model} \times d_{ff}}$, $W_2\in \mathbb {R}^{d_{ff}\times d_{model}}$, $b_1\in \mathbb {R}^{d_{ff}}$ and $b_2\in \mathbb {R}^{d_{model}}$ are trainable parameters. *d*_*ff*_ denotes the hidden dimension of the MLP. The final output of the encoder block is computed by applying another residual connection followed by layer normalization


(8) \begin{eqnarray*} O = LN(\mathrm{MLP}(N) + N). \end{eqnarray*}

This encoder block design can be stacked multiple times to generate high-level representations. After the last encoder block, the output is fed into a linear classifier. Since *O* is a sequence of representations, it has to be transformed into a single vector which can be used with a fully-connected layer. The first option is to simply concatenate all vectors of the sequence into a single long vector. This is sub-optimal since it is dependent on the sequence length producing non-fixed-sized outputs. The second option is to take the mean along the sequence dimension across all sequence elements. The third option is to prepend a special classification token <*cls* > to each sequence and solely use it for classification. Since the 0th element of the sequence will always be used for classification, the network will learn to compress all information into this vector that is relevant for classification. To summarize, the final sequence of representations can be derived by


(9) \begin{eqnarray*} O_{red} &= W_{red}\cdot \text{concat}(O_1,..., O_n) \quad &\text{(concatenation)} \end{eqnarray*}

(10) \begin{eqnarray*} O_{red} &= \frac{1}{n}\sum _{i=1}^{n}O_i \quad &\text{(mean reduction)} \end{eqnarray*}

(11) \begin{eqnarray*} O_{red} &= O_0, \quad &\text{(cls token)} \end{eqnarray*}

where $W_{red}\in \mathbb {R}^{d_{model}\times (n\cdot d_{model})}$ is a trainable parameter to reduce the concatenation into a single vector of size *d*_*model*_ again. Therefore, *O*_*red*_ is of size *d*_*model*_ with every method of reduction. The actual classification is then performed by a linear classifier


(12) \begin{eqnarray*} LC = O_{red}W_1 + b_1, \end{eqnarray*}

where $W_1\in \mathbb {R}^{d_{model} \times m}$ and $b_1\in \mathbb {R}^{m}$ are trainable parameters and *m* denotes the number of output classes. For the choice of hyperparameters we refer to (18,45). Further, the memory consumption of the embedding and the transformer are important factors to be taken into account to meet the memory capacity constrains of GPUs. An overview over the hyperparameters that we evaluated can be found in Table 2.

**Table 2.** Hyperparameters for our transformer encoder models

Parameter	*d*_*model*_	encode block	*d*_*ff*_	reduction	dropout
Values	64/128	1/2/3/4	256/512	mean/cls	0.0/0.1

An overview over the hyperparameters used for our transformer encoder models. Slashes indicate the different options that were evaluated for the hyperparameters.

### Multi-level classification

While models like DeepMicrobes perform well at a single taxonomic rank, it is often required to perform classification at multiple taxonomic levels. For example, if a read cannot be classified at species level with sufficiently high confidence, the user would still be interested in the genus-level classification. In order to predict at multiple ranks, we can add more classification layers after the backbone of the model. The backbone constitutes the part of the model that generates an encoded representation from the input data. Each classification head operates independently from the other heads. Let $x_b\in \mathbb {R}^{dim_{b}}$ be the output of the backbone for a single input instance *x* with dimension *dim*_*b*_. Let furthermore *M* = (*M*_1_, ..., *M*_*N*_) denote the number of output classes at *N* different taxonomic levels. Then each classification head *H*_*i*_ produces an output


\begin{eqnarray*} \hat{y}_i = \mathrm{MLP}_i(x_b), \end{eqnarray*}

where $\hat{y}_i\in \mathbb {R}^{M_i}$ denotes the unnormalized class scores over the output classes *M*_*i*_. *MLP*_*i*_ is the corresponding multi-layer-perceptron with input and output dimension *dim*_*b*_ and *C*_*i*_, respectively. The MLP can exhibit an arbitrary amount of hidden layers of suitable dimensions.

Rojas-Carulla *et al.* (14) augmented this basic idea by introducing interconnections between the classification heads. This has the advantage that higher-level layers can directly inform lower-level layers about their prediction. Let $x_b\in \mathbb {R}^{dim_b}$ denote the representation of input *x* again. The prediction on level *i* of the multi-headed interconnected classification layer is described by


**Figure 4.**
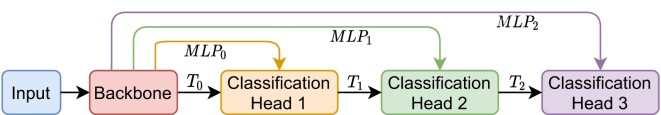
Multi-level interconnected classification heads. Each classification head uses the representation generated by the model backbone to perform prediction. Additionally, higher-level predictions inform lower levels about their prediction by linearly transforming their output to match the output shape of the lower level.

(13) \begin{eqnarray*} \hat{y}_i = \mathrm{MLP}_i(x_b) + T_{i-1}\hat{y}_{i-1}, \end{eqnarray*}

where $\hat{y}_i\in \mathbb {R}^{M_i}$ denotes the prediction and MLP_*i*_ the respective MLP on level *i*. To use the prediction $\hat{y}_{i-1}$ from the previous level, its dimension is transformed using a trainable linear layer $T_{i-1}\in \mathbb {R}^{M_i\times M_{i-1}}$ and *T*_0_ denotes the zero matrix. Our proposed classification scheme is illustrated in Figure 4.

During training, the classification heads are jointly trained. Therefore, the loss function needs to be adapted to account for the newly introduced heads. Let $Y=(y_1,...,y_N)$ and $\hat{Y}=(\hat{y}_1,...,\hat{y}_N)$ be the ground-truth taxa and the predicted taxa on each taxonomic level, respectively. For the following, we assume that a cross-entropy loss is used at each level *i*. It is defined as


(14) \begin{eqnarray*} CE_i(\hat{Y_i}, Y_i) = - \sum _{k=1}^{C_i}Y_{ik}\cdot \log (\hat{Y}_{ik}). \end{eqnarray*}

Note that any loss function suitable for multi-class prediction could be used instead. A problem that is reintroduced by multi-level classification is class imbalance. While it is possible to establish class balance at a single taxonomic level by sampling an equal proportion of reads per class during read-generation, the same can not be achieved for multiple levels. Consider a balanced dataset on the species level. When performing genus level prediction for this dataset, it is likely that the species will not be assigned to the genera in equal proportion. The same pattern will continue for higher levels. To avoid this issue weighting factors $(w_1,...,w_N)$ with $w_i\in \mathbb {R}^{C_i}$ are introduced. Each $w_i$counteracts over- or under-represented classes on the respective level *i*. The cross-entropy loss function would be then altered to


(15) \begin{eqnarray*} CE(\hat{Y}, Y) = \sum _{i=1}^{N}w_{i,y_i} \cdot CE_{i}(\hat{Y_i}, Y_i). \end{eqnarray*}

Each $w_i$needs to be calculated once on the training data before training. The weights $w_i$are the normalized inverse counts of the of the classes *C*_*i*_. In practice, interconnected multi-headed classification should perform at least equally to normal multi-headed classification. It can potentially perform better since higher-level predictions can inform lower levels about their decision.

### Training

All models were trained using the Adam optimizer (46) with an initial learning rate of 0.01. When training a model with sparse embedding gradients, the weights of the embedding were separately optimized by SparseAdam, a PyTorch (47) implementation of Adam that is suited for sparse gradients. Since we are targeting classification with more than two classes, we used cross-entropy loss as cost function as seen in Equation 14. We tracked the loss on the validation data to determine the training progress and prevent overfitting. For all models, we used a batch size of 2,048. Species- and genus-level models were trained for a maximum of 300,000 or 500,000 steps. Early stopping was used when the model did not improve on the validation data for more than 50,000 steps. The training data was read sequentially from the FASTA files through a custom implementation of the IterableDataset class from PyTorch. This drastically reduces RAM usage since only data required for the current batch needs to be held in memory. We used a PyTorch Dataloader with 8 to 16 worker processes and a pre-fetch factor of 2 to saturate the GPU during training. Training inputs are raw DNA reads which are subsequently transformed using custom implementations of PyTorch transforms objects. This includes tokenization into characters, *k*-mers or sub-words like byte-pair econding (BPE). Furthermore, naive hashing and locality-sensitive hashing (LSH) are also implemented as transforms since they operate on *k*-mer indices.

We implemented LSH as a Cython extension (48). In this manner, we operated on C-strings and could directly call the C++ implementation of MurmurHash3 (49) for hashing. We also added the option to use mixed-precision computations for an additional speedup and lower memory requirements during training and inference. Major frameworks already include respective modules to easily add mixed-precision capability. In our case, we use the AMP (automatic mixed-precision) module shipped with PyTorch.

### Inference

Similar to training, DNA reads constitute the input during inference. Here, the same set of transformations used in training were applied. We used paired-end reads during testing to improve resolution. Before inference, paired-end sequences were interleaved. The model then performs the prediction separately on each of the sequences. Afterwards, the results were component-wise averaged before applying the softmax function. To saturate the GPU during inference, we used the dataloader with up to 80 threads.

### Evaluation metric

In order to evaluate the efficacy of our model, we employ a range of quantitative measures. For assessing the test data, we utilize precision and recall metrics defined as:


(16) \begin{eqnarray*} \text{ Precision}_{read} = \frac{\text{\# reads classified correctly}}{\text{\# reads classified}} \end{eqnarray*}

(17) \begin{eqnarray*} \text{ Recall}_{read} = \frac{\text{\# reads classified correctly}}{\text{\# reads}} \end{eqnarray*}

Moreover, in our study, we incorporate the L2 distance metric and employ the Linear Discriminant Analysis Effect Size (LEfSe) (41) for conducting differential abundance analysis. The LEfSe tool utilized in our analysis was obtained from the Huttenhower Galaxy Server (available at http://galaxy.biobakery.org/). For the Kruskal-Wallis test, we set a significance threshold of 0.05, and the logarithmic Linear Discriminant Analysis (LDA) score threshold for identifying discriminative features was set at 2.0. Additionally, we applied per-sample normalization of the sum of the values to 1 million.

### Hardware and software

Taining and benchmarking were conducted on a standalone workstation featuring two Intel Xeon Gold 6238 processors with 22 cores each and 188GB of RAM. Additionally, it contained an NVIDIA Quadro GV100 GPU with 32GB HBM2. We used Python 3.8 for training and experiments. All models were run using PyTorch 1.8.1 and CUDA 11.2. The byte-pair encoding was generated using the BPE model from the huggingface tokenizer library. We used Jellyfish (50) to create *k*-mer vocabularies.

## RESULTS

### Embeddings and tokenizations

#### Performance of *k*-mer-based models

We tested our transformer-encoder-based model on *k*-mer tokenization in the following referred to as *Vocab*. *k*-mer size is a key factor for performance. Experiments on the LSTM-based architecture have shown that larger values of *k* are desirable while accuracy drops rapidly for lower values of *k*. Our training was performed for *k* ranging from 7 to 13.

**Figure 5.**
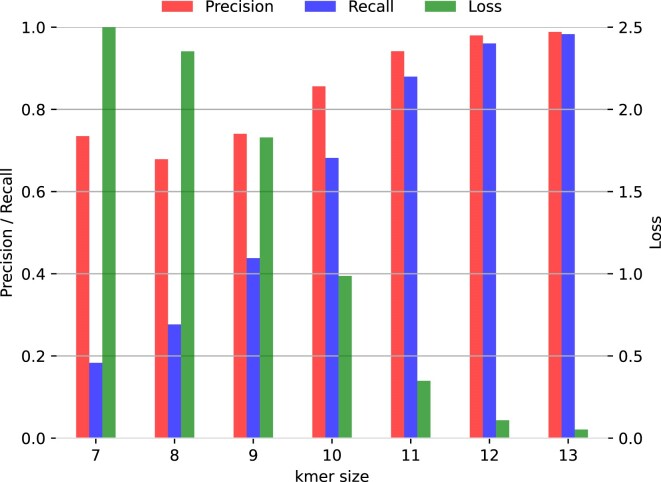
Performance of *Tf Vocab* for increasing *k*-mer size. Precision, recall and loss for increasing values of *k* for the *Tf Vocab* architecture are displayed. Values were measured during training on the validation data based on the *HGR UMGS* dataset.

In contrast to Liang *et al.* (16), we were able to evaluate *k* = 13 for our transformer by using mixed-precision, sparse embedding gradients, and a smaller model dimension *d*_*model*_ = 64. The results are summarized in Figure 5. Like DeepMicrobes, *Vocab* exhibits low precision and recall on the validation set for *k* < 11. The best-performing model uses *k* = 13. We suspect that larger values of *k* would be beneficial, especially when dealing with more classes. Unfortunately, increasing *k* also increases memory consumption, e.g. *k* = 14 exhausts 32GB of GPU memory and could therefore not be tested on our GPU. Hence, for further experiments we focused on the *k* = 12 and *k* = 13 model. Furthermore, *Vocab* model with *k* = 12 optimized for multi-level classification (*Vocab ML*) was trained. Though, *Vocab ML* showed a lower precision and recall compared to *Vocabk* = 12 and *k* = 13 for the genus prediction. Precision and recall for *Vocab* *k* = 12 and *k* = 13 and *Vocab ML* are summarized in Table 3.

**Table 3.** Genus-level performance for different embedding schemes

Scheme	*k* / *b*	Data	Prec	Recall	Loss	Time (h)
*LSH*	15 / 2^22^	Train	0.979	0.953	0.125	27
Val	0.969	0.938	0.185			
*LSH*	15 / 2^23^	Train	0.984	0.966	0.094	25
Val	0.984	0.966	0.094			
*Hash*(*q* = 6)	13 / 2^22^	Train	0.977	0.949	0.137	18
Val	0.977	0.950	0.137			
*Vocab*	12	Train	0.980	0.961	0.103	20
Val	0.980	0.970	0.090			
13	Train	0.989	0.980	0.054	10	
Val	0.989	0.983	0.053			
*Vocab ML*	12	Train	0.972	0.952	2.920	43
Val	0.970	0.950	2.942			

Performance of the embedding schemes in combination with the transformer model on the genus-level. We only included the best performing variants for each scheme. Precision, recall and loss of the model on the training and validation set as well as the training time of each model are reported. *b* represents the number of buckets. All models, except for *LSH* (*k* = 15, *b* = 2^23^) with 420,000, the *Vocab ML* with 1,000,000 and the Vocab (*k* = 12) with 500,000, are learned for 300,000 epochs.

#### Performance of character-level tokenization and byte-pair encoding

We tested character-level tokenization and byte-pair encoding schemes with our proposed architecture. For character-level encoding each character is represented by an embedding according to its index in the vocabulary. Since the employed embedding only had few entries, namely the 4 bases A, C, G,and T, we experimented with deeper architectures featuring more encoder blocks. We tested an architecture with an embedding size of 128 and 6 encoder blocks. Additionally, we used an embedding of size 64 and increased the number of encoder blocks even further up to 12 blocks. The best result was achieved with a dimension of 128 and 6 encoder blocks at a precision of 0.764 and a recall of 0.265 on the *HGR UMGS* validation data. Compared to *k*-mer-based approaches the performance is drastically lower. Naturally, with longer substrings, the vocabulay size increases allowing for more detailed pattern recognition (28).

For byte-pair encoding we tested similar hyperparameters as the *k*-mer-based networks with a vocabulary size of 2^22^ and 2^23^. In contrast to tokenization based on *k*-mers, subword-level tokenization produces much shorter sequences. This allowed us to increase the number of transformer encoder blocks up to 4 due to the reduced memory requirements. The best model with 1 block achieved a precision and recall of 0.891 and 0.643 on the *HGR UMGS* validation data. This is also significantly worse compared to the *k*-mer-based variant summarized in Table 3. We attribute the reduced performance to the fact that tokens in byte-pair encoding do not overlap. Therefore, single nucleotide changes may lead to entirely different subword tokens. Additionally, subword tokens do not allow for creation of local canonical representations of a read as it is the case with *k*-mers. As a result, we use the *k*-mer driven approaches for further analysis.

#### Performance of LSH and hash embeddings

In contrast to Georgiou *et al.* (15), we propose a less memory-restrictive usage of LSH and hash-embeddings. The memory reduction schemes should rather be used to enable larger *k*-mer sizes than heavily reducing the memory footprint. Here, the transformer-encoder-based on LSH or hash embedding schemes are referred to *LSH* and *Hash* respectively. For *LSH* we opted for *b* = 2^22^ or *b* = 2^23^ buckets and *k* = 15. For hash embedding we used a bucket size of *b* = 2^22^, *q* = 6 hash weights per *k*-mer and a *k*-mer size of *k* = 13. The results of the respective schemes at genus-level are summarized in Table 3. Both versions of *LSH* achieve reasonable performance on the validation data. *LSH* with only *b* = 2^22^ buckets seems to generalize worse than for *b* = 2^23^ as indicated by the validation loss. The *Hash* performance is located in-between the variants of *LSH*. Overall, *LSH* and *Hash* show better precision and recall compared to their character-level and byte-pair counter parts. Though, the *k*-mer embedding approaches without compression show an overall better performance even though having a lower *k* compared to *LSH*. Therefore, for the following genus analysis steps *Vocab* *k* = 12 and *k* = 13 were used and are referred to as *MetaT k12* and *MetaT k13*.

#### Species prediction

For species prediction *Vocab* *k* = 12 and *k* = 13 were selected due to their good performance at genus level. Further, *Vocab ML* can also classify species sequences. Table 4 shows precision, recall, loss and training time for the three models. Like for genus prediction, *Vocab* *k* = 13 shows the best performance on both training and validation set, followed by *Vocab ML* and *Vocab*  *k* = 12. As a result, we used *MetaT k12* and *MetaT k13*, due to their good performance and good comparability to DeepMicrobes for further species analysis.

**Table 4.** *Vocab* embedding species-level performance

Scheme	*k*	Data	Precision	Recall	Loss	Time (h)
*Vocab*	12	Train	0.848	0.475	1.828	20
		Val	0.866	0.537	1.620	
	13	Train	0.914	0.721	0.962	16
		Val	0.909	0.703	1.033	
*Vocab ML*	12	Train	0.909	0.516	2.920	43
		Val	0.905	0.515	2.942	

Performance of the *Vocab* embedding schemes in combination with the transformer model on the species level. We only included the best performing variants for each scheme. Given are the precision, recall and loss of the model on the training and validation set as well as the training time of each model. Both *k* = 12 and *k* = 13 are trained for 500,000 epochs while ML is the same model as seen in genus with 1,000,000 epochs.

### Performance of MetaTransformer on test data

#### MOCK community analysis

In the following we analyze the performance of different embedding schemes for the genus estimation on the *BenchmarkMock* dataset. The best performing models during training, *MetaT k12* and *MetaT k13*, were tested against current classification tools for genus and species classification on this dataset. As comparison metric, the L2 Norm distance is used.

**Figure 6.**
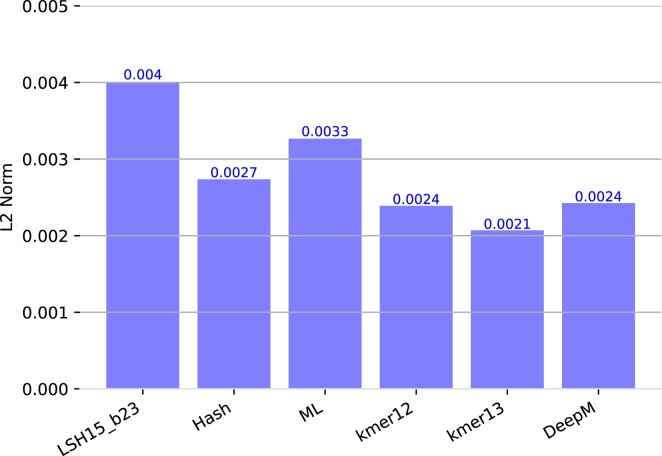
Average L2 Norm of the genus abundance prediction error for different embeddings on the *BenchmarkMock* dataset. DeepMicrobes is used as a reference.

**Figure 7.**
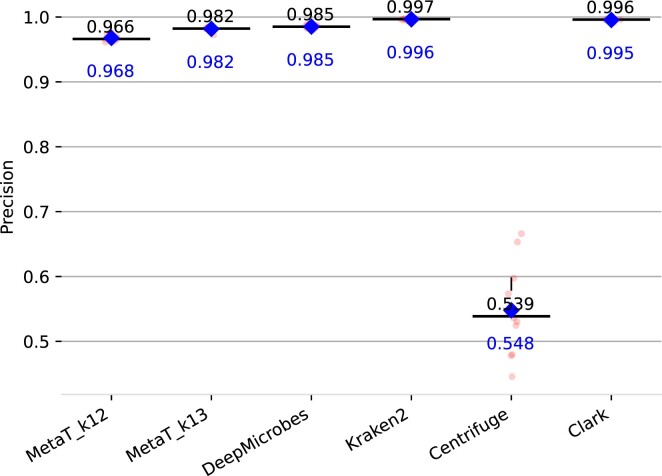
Precision of the genus abundance prediction for different classification tools on the *BenchmarkMock* dataset. Median is colored in black and mean in blue.

**Figure 8.**
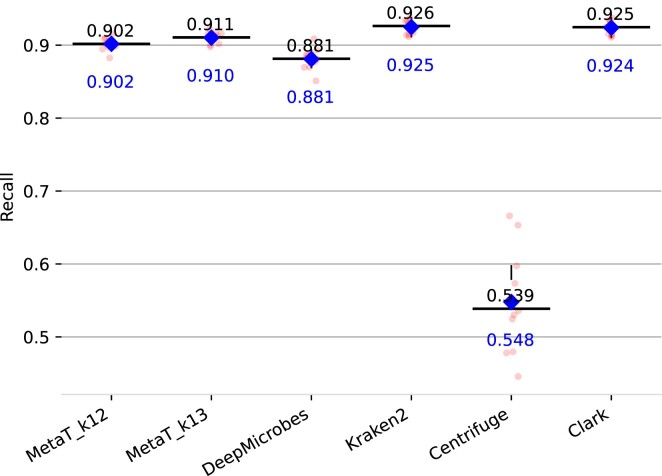
Recall of the genus abundance prediction for different classification tools on the *BenchmarkMock* dataset. Median is colored in black and mean in blue.

The results of our different embedding schemes for genus abundance estimation are summarized in Figure 6. Best performing model for genus prediction was again *Vocab* *k* = 13 model with an average distance of 0.00207, followed by the *k* = 12 variant (0.00239), and DeepMicrobes (0.00242). *LSH* with *k* = 15 and 2^23^ buckets performed worst with 0.00400 even though exhibiting a good performance during training. All in all, the results on the *BenchmarkMock* dataset reflect the performance seen on the validation set. Only *Vocab* shows a lower distance than DeepMicrobes underlining the importance of a suitable embedding scheme for model performance. For genus classification of the other tools we measure the precision and recall as previously defined. All tools are build on the same genomes we used for training our model. Our transformer model outperformed DeepMicrobes in recall (0.911 vs 0.881) yet has a slightly lower precision (0.982 vs 0.985) for *k* = 13. Nevertheless, both deep learning models were outperformed by Kraken2 and CLARK. Centrifuge scored the lowest score. Note, the precision and recall are equal for centrifuge, because every read was classified. Precision and recall results are summarized in Figures 7 and 8.

#### MAG analysis

**Figure 9.**
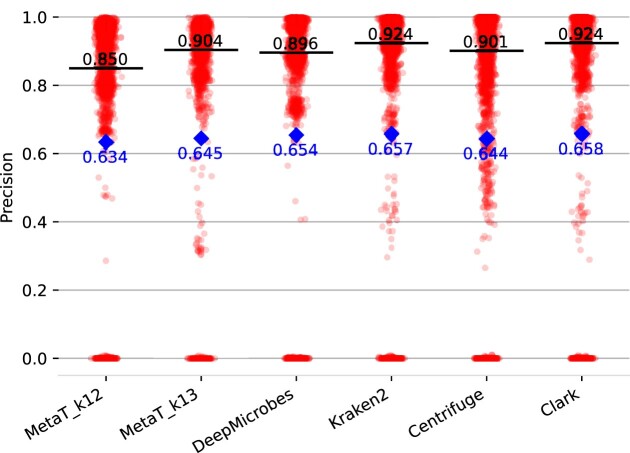
Precision of the species abundance prediction for different classification tools on the *BenchmarkGut* dataset. Median is colored in black and mean in blue.

**Figure 10.**
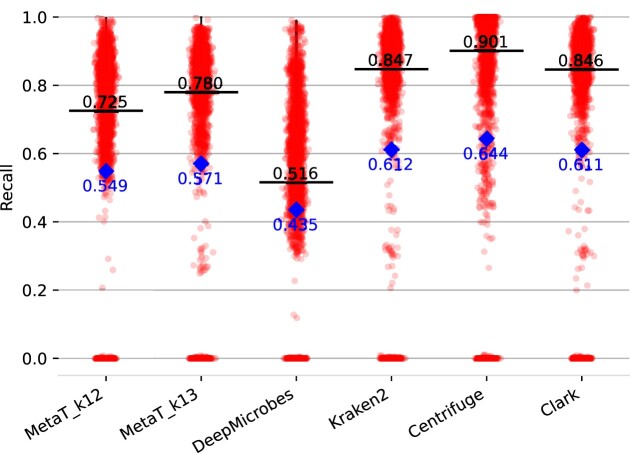
Recall of the species abundance prediction for different classification tools on the *BenchmarkGut* dataset. Median is colored in black and mean in blue.

The *BenchmarkGut* dataset consist of 3269 gut derived high quality MAGs. For each MAG the closest corresponding species was calculated as previously described. We use this dataset to evaluate the species prediction capabilities of our approach. The precision and recall for species prediction are summarized in Figures 9 and 10. For the other tools custom databases and taxonomic trees were generated to use the same species genomes as the deep learning approaches. Our approach outperforms DeepMicrobes in recall and precision (0.904 versus 0.896 and 0.780 versus 0.516) for *k* = 13. Though, it performs slightly worse than Kraken2 in prediction. The best tool overall for precision is CLARK and Kraken2 (0.924) while centrifuge scores the best recall (0.901).

#### Absent species

**Figure 11.**
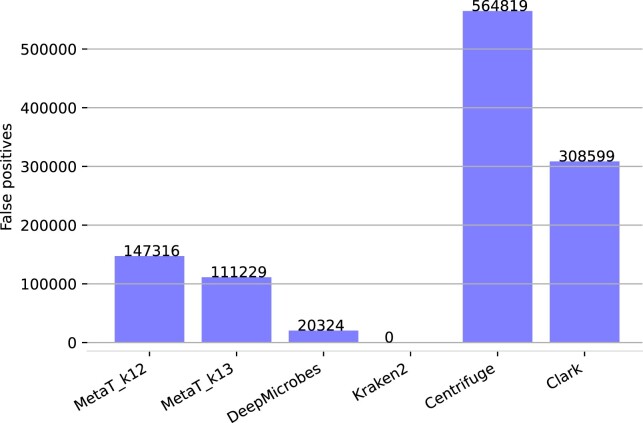
Total number of misclassified reads for different classification tools on the *AbsentSpecies* dataset.

Another important problem for metagenome analysis are absent species in the database leading to high false-positive rates. Therefore, the *AbsentSpecies* dataset containing 121 species absent from all databases was generated (16). Figure 11 shows the total accumulated abundance of several classifier. In this experiment, Kraken2 outperformed all classifiers with 0 false positives followed by DeepMicrobes and our approaches (20,324 versus 111,229 and 147,316). Centrifuge mapped every read hence it performed worse with an abundance of 564,819.

### Differential species abundance analysis on real-world data

We analyzed 106 sequenced gut metagenome fecal samples using our MetaTransformer model (*MetaT k13*). The samples are part of iHMP (39) and were collected from subjects with ulcerative colitis (uc), Crohn’s disease (cd) and healthy specimens. We want to test if newly discovered genomes are correlated with IBD and if our model is suitable for real world data. Therefore, we calculated abundances via our model and determined differential abundant species using LEfSe.

**Figure 12.**
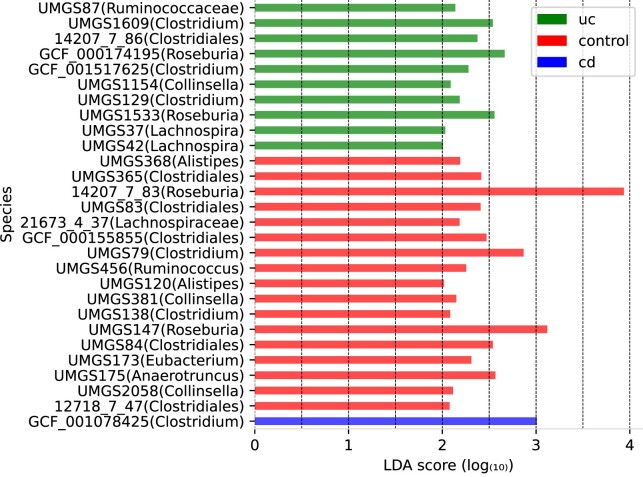
LDA score of significant differentiated species. LDA score of every species with significant (Kruskal-Wallis *P* < 0.05 and LDA score > 2) difference between two or more groups. cd is representative for Crohn’s disease, uc for ulcerative colitis and control for healthy subjects. In brackets is the closest taxonomic classification for each species.

After the Kruskal-Wallis test and LDA calculation, 28 species showed a differential abundance over the different groups. Especially, we found differential abundance in *Alistipes*, *Anaerotruncus*, *Clostridium*, *Collinsella*, *Eubacterium*, *Lachnospira*, *Roseburia*, *Ruminococcus* and unclassified species of the order *Clostridiales*. All species and their LDA scores are summarized in Figure 12. From these 28 species, five (14207_7_83, GCF_000155855, GCF_000174195, UMGS365, UMGS368) displayed differential abundance in DeepMicrobes. Further, most found genera are identical between both tools, though DeepMicrobes found differences in *Prevotella, Coprococcus, Alphaproteobacteria, Anaeromassilibacillus* and *Bacteroides*, while MetaTansformer found variations in *Anaerotruncus, Collinsella* and *Eubacterium*.

Overall, the identified species indicate a correlation to healthy, uc and cd subjects as reported for their genus/order. For example UMGS175 (*Anaerotruncus*) shows a decreased abundance of cd compared to the control and uc sample, which is described by Zhuang *et al.* (51) for this genus. UMGS456 of genus *Ruminococcus* expresses a decreased abundance in both cd and uc, compared to the control, following the same pattern as outlined in the literature (51,52). Though, species-specific patterns like lower abundance of *R. obeum, R. callidus* and *R. lactaris* in IBD (53), higher abundance in cd like *R. gnavus* (53–56) or a loci specific abundance with a lower abundance of *Ruminococcus* in the ileum and a higher abundance in the colonic region are characterized (56).

**Figure 13.**
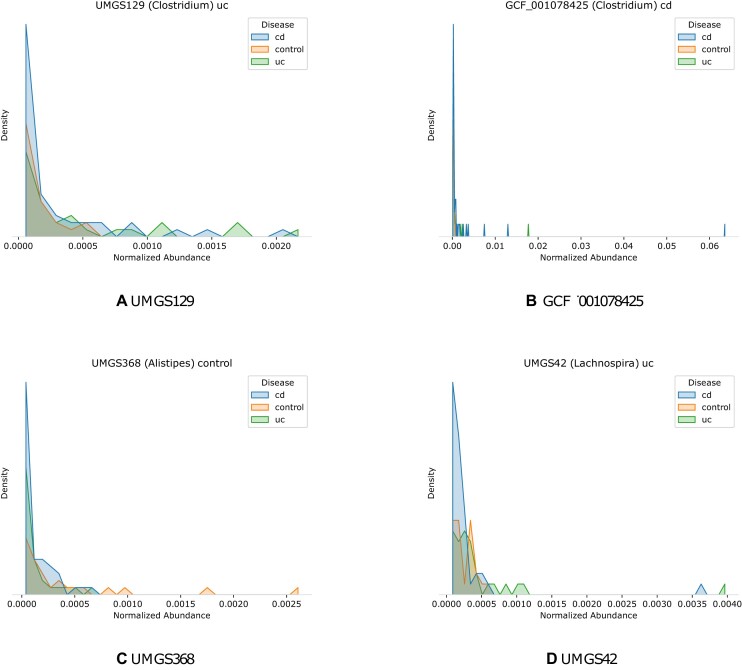
Normalized abundance density of the different groups for species UMGS129, GCF_001078425, UMGS368 and UMGS42. Crohn’s disease is represented by cd, ulcerative colitis by uc and healthy subjects by control. The abbreviation after the genus is the significant group determined by the LEfSe.

*Clostridium* in general shows a lower abundance in IBD specimens (52,56). Nevertheless, fluctuations have been shown for different species like a higher abundance in cd for *C. difficile*, a higher abundance in uc for *C. clostridioforme* (53), higher abundance in healthy subjects for *C. innocuum and C. ramnosum* (55) and a lower abundance in healthy subjects for *C. leptum* (53,55,56) We estimated a higher abundance on uc compared to cd and healthy subjects for UMGS129 (Figure 13A) while GCF_001078425 (Figure 13 B) has the highest abundance for cd followed by uc. Also, species like *C. innocuum* and C. ramnosum are described with the highest abundance for the control group (55), underlining the species specificity of differential abundances in the bowel flora.

UMGS368 (Figure 13 C) belongs to the genus *Alistipes* and showed a higher abundance in healthy specimens compared to IBD subjects. The same results can be seen in previous studies for different species of *Alistipes* like *A. shahii, A. finegoldii* and *A. putredinis* (39,57) as well as in the DeepMicrobes study (16) showing a comparable abundance for the same species.

Two species of *Lachnospira* show a significant difference between the groups. In both cases, uc has the highest mean abundance (Figure 13D). In general, *Lachnospira* shows a higher abundance in uc and cd compared to control subjects (52,58). Though, differences between species of *Lachnospira* with a lower abundance in uc and cd are reported as well (56,59,60).

All in all, we found significant differential abundance in IBD and control specimens for newly discovered species. These new species might provide insights of the development as well as the diagnosis and treatment IBD patients. The abundant densities for every significant differentially abundant species are listed in the Supplement (Supplementary Figure S2–S29).

### Training and inference speed

#### Training

Model training times are dependent on the type of embedding scheme, number of learning steps, performance booster (AMP and sparse matrix), model size, as well as training level like genus or species. Tables 3 and 4 show the training times of different embeddings for genus and species abundance prediction. The fastest trained genus model was *MetaT k13* with around 10 hours training time for 300,000 steps. With over 43 hours and 1,000,000 steps the multi-level variant took the longest, though it learned genus and species prediction simultaneously. For species prediction, *MetaT kmer13* with 16 hours was again the fastest, 4 h faster than the 12 *k*-mer variant with the same 500,000 steps.

#### Inference

Due to space limitations for the larger datasets *BenchmarkMock* and *BenchGenus*, both were split into multiple smaller files and processed successively. For the smaller species sets multiple files were processed simultaneously. On the *BenchmarkMock* dataset *MetaT k13* outperforms DeepMicrobes with an average speedup of 5.7× for genus and 1.4× in species detection. *MetaT k12* on average acquires a speedup of 5.0× for genus and 2.0× for species detection. All speedups presented are given on the basis of million read-per-minute (MRPM). The different speedups of the transformer models are based on *k*-mer 13. *MetaT k13* has a smaller embedding dimension and feed-forward dimension than *MetaT k12*, while the structure is equal. Therefore, calculation is faster which is also seen in the faster training times. However, with the smaller size *MetaT k12* can process more species files simultaneously. Furthermore, model loading times are longer for the *MetaT k13* model. The speedup and times for both benchmark datasets are summarized in Table 5 Another important factor is data preprocessing. DeepMicrobes needs a lengthy transformation step before working on the data. These steps are run via multiple programs using single and multi-threaded parts. For the *BenchmarkMock* dataset each of the ten MOCK samples took around 25 min to complete which is about half the time needed for the evaluation only. Furthermore, after the preprocessing steps the size of each file inflates 6-fold. The preprocessing embedding steps were ignored in the comparison. MetaTransformer calculates the embeddings on-the-fly using multi-threaded CPU processing and GPU accelerated computing, greatly reducing space consumption and time.

**Table 5.** Species and genus-level inference speed for *BenchmarkMock*

	Level	*MetaT k12*	MetaT k13	DeepMicrobes
Time (s)	genus	517	458	2622
	species	1396	1604	2833
MRPM	genus	1.16	1.31	0.23
	species	0.43	0.37	0.21
Speedup	genus	5.1	5.7	1
	species	2.0	1.5	1

Inference speed for genus and species-level of *MetaT k12*, *MetaT k13* and DeepMicrobes on the *BenchmarkMock* dataset. The results are averaged over all 10 samples.

## DISCUSSION

Taxonomic classification tools are often based on alignment-free mapping approaches enabling the analysis of millions of reads with a high accuracy. Deep learning has shown its potential in a variety of problems on sequence-based data (12,13,28–30). Therefore, new classification approaches were explored using deep learning with good results on genus and species prediction even on non-curated databases (16). However, deep learning-based methods come with a high computational cost and lower throughput, especially when using bi-directional LSTMs and compared to state-of-the-art conventional metagenomic classifiers (8,16,61). Yet, with new advancements in deep learning, like complex transformer models (18) such as BERT (11) for language modeling, new approaches have the potential to improve metagenomic classification tasks.

In this work. we introduced MetaTransformer, a transformer-based classification algorithm for genus and species levels. We were able to train the network within 9 − 16h and speed up inference time by up to 5 times compared to the LSTM approach. Furthermore, we can match the classification performance of a previous approach, DeepMicrobes, in genus prediction and outperform it at species-level prediction on our test setups.

One limitation of our approach is the updateability. Adding new species/genera would in principle require retraining of the entire network. Nevertheless, it is possible to retrain on the previous model while adding new species, as seen in incremental learning (62,63) or to retrain with an entirely new set of genomes in a transfer learning manner (64). Such approaches would greatly reduce the computational cost of changing the underlying database and need to be further investigated.

Unfortunately, we still underperform in terms of several metrics compared to conventional alignment-free approaches. Here, a more sophisticated approach like DNABERT needs to be explored and tested for metagenomics. Furthermore, more diverse input data for intensive training and testing is needed. Nevertheless, with such sophisticated models the throughput and learning times typically increase from the additional complexity. Also classification would take considerable more time. We tested the runtime of BERTax (65), a classifier based on BERT, resulting in an average throughput of 37 reads per second on average tested on the *BenchmarkMock* dataset, which is 500 times slower than our approach.

We also incorporated memory reducing LSH and Hash-embedding schemes into our new architectures. Instead of solely focusing on the reduction of memory, we used them to enable larger *k*-mer sizes up to *k* = 15. Although we could not observe any significant improvements over the *k*-mer-based *Tf Vocab* version, both schemes performed comparably. When using *Tf Lsh* with a slightly restricted amount of buckets (*b* = 2^22^), reasonable performance can be achieved while maintaining a relatively low memory footprint. The investigation of character- and sub-word-level tokenization methods showed that they both performed significantly worse when compared to *k*-mer level encoding. Byte-pair-encoding suffers from undetectable single nucleotide changes since subword tokens are generated in a consecutive and not in an overlapping manner.

To demonstrate the applicability of our approach, we analyzed a real-world metagenomic gut dataset with focus on IBD (39). We discovered potential candidate species for characterizing uc, cd and healthy specimen in the uncultured species, validating the importance of such datasets. Our results and the current research show a species specific interaction between the microbiome and IBD (39,53,66), though a detailed view is still missing. Therefore, more research must be conducted in the use of uncultured species and effect on the overall gut health possibly decoding the missing links in the IBD emergence.

With the rapid growth of computational power and advances in deep learning, new approaches might become a viable strategy for taxonomic read classification alongside established alignment-free methods, especially for high-dimensional data and multiple analysis steps on big data problems. Hence, further research in the field is needed.

## Supplementary Material

lqad082_supplemental_file

## Data Availability

MetaTransformer with the different embedding schemes as well as further information is available at https://zenodo.org/badge/latestdoi/584424427 and https://zenodo.org/badge/latestdoi/585097144.
