# Supplementary material for: MetaTransformer: deep metagenomic sequencing read classification using self-attention models
Source: NAR Genom Bioinform. 2023 Sep 11;5(3):lqad082. doi: 10.1093/nargab/lqad082 (PMC10495543; doi:10.1093/nargab/lqad082)
Supplement: lqad082_supplemental_file [file lqad082_supplemental_file.pdf]

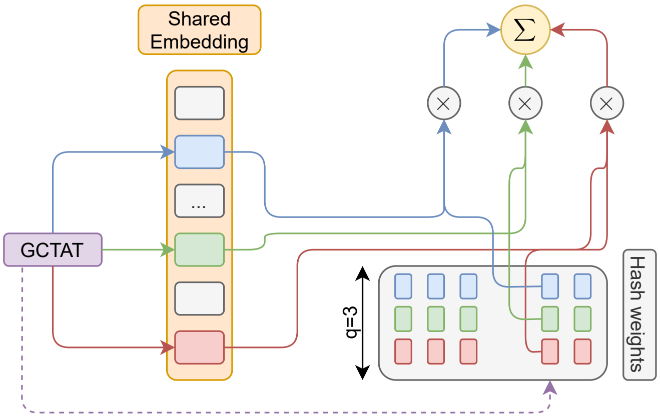

Supplementary Figure 1. Hash-embedding scheme: An input k-mer is initially hashed with $q=3$ different hash functions. Afterwards the selected shared embeddings are aggregated based on the set of trainable weights for the respective k-mer.

SUPPLEMENT

Embedding Schemes

LSH Embedding Overall memory consumption can be reduced by applying an additional embedding step (1). Locality-sensitive hashing (LSH) (2) is a potent method to drastically reduce the input size while retaining relevant information. The intend of LSH is to find similar objects (here k-mers) in a set by means of a generated hash signature per object. The method ensures that objects with similar signatures end up in the same hash bucket with a high probability. Here, the used LSH algorithm is based on minhashing (3). This scheme provides precise control over the number of used buckets b while allowing for arbitrary choices of the k-mer length.

Hash Embedding Another hash based method provides a set of shared embeddings for all k-mers. Out of these embeddings, a k-mer can construct its own embedding based on weights learned during the training process. Initially, each k-mer is mapped to a unique index by a function $K: L^k \rightarrow \mathbb{Z}_{|L|^k}$ where L^k is the vocabulary of k-mers. In our case, this is achieved by a predefined vocabulary that maps each k-mer to a unique index. Subsequently, each k-mer is hashed using the q hash functions $H_1,...,H_q : \mathbb{Z}_{|L|^k} \rightarrow \mathbb{Z}_s$ where each hash function H_i maps the k-mer index to the set of integers modulo s. This corresponds to one entry from the set of shared embeddings.

Let $S \in \mathbb{R}^{s \times d}$ denote a shared embedding matrix and $W \in \mathbb{R}^{|L|^k \times q}$ a trainable weight matrix. Here s denotes the size of the shared embedding, d the size of the embedding vectors and q the number of employed hash functions. To generate a unique embedding for a k-mer x, it uses its own set of q weights $L(x) = W_{K(x)}$ to calculate the weighted sum of the q embeddings. In this manner, a unique embedding can be produced even though dissimilar k-mers use the same representation from the set of shared embeddings. The final embedding of a k-mer x is given by $\sum_{i=1}^{q} L(x)_i \odot S_{H_i(K(x))}$.

The proposed scheme is illustrated in Supplementary Figure 1.

Byte-Pair Encoding Since the number of sub-word tokens is a user-controllable parameter, the resulting memory footprint of the embedding can be adapted to the available resources. Additionally, sub-words do not overlap, like it is the case for k-mers, resulting in usually significantly shorter sequences further reducing memory consumption. Byte-pair encoding was implemented using the BPEModel provided by the huggingface tokenizers library (4). For training, we used all genomes from the *HGR UMGS* training dataset. Specifically, we extracted elements of 150bp length by applying a sliding window over each genome. The sliding window uses a stride of 130. Therefore, each window overlaps by 20bp to capture sub-words located at the edge of two windows. Each element is additionally transformed into its canonical representation. Afterwards, we randomly shuffled all lines and used approximately 1.5 gigabyte of sequence data to train the BPEModel. The BPEModel treats every line as a single token. We decided to use tokens of length 150bp since it allows the model to learn long tokens while keeping a reasonable memory footprint during the generation process. We set the target vocabulary size to 2^{22} .

Sparse Embedding Gradients Using a sparse gradient the input dimension gets reduced while preserving the sparse structure of the data (5). In addition, fewer operations are needed for the weight update during backpropagation which speeds up the training process. Thus, most popular deep learning frameworks, e.g., TensorFlow or PyTorch, offer embeddings where the gradient is stored in a sparse format during backpropagation. Popular choices for the sparse storage formats include coordinate-list (COO) and compressed sparse row/column (CSR/CSC).

Layer Normalization

In our case, each element of the sequence exhibits a mean of zero and a variance of one after normalization. Given our sequence $Z' \in \mathbb{R}^{n \times d_{model}}$ each row Z'_i is normalized as follows. First the mean and variance of the sequence element Z'_i is calculated. Afterwards it is normalized to mean zero and variance one by

$$\hat{Z}_i = \frac{Z'_i - \mu_i}{\sqrt{\sigma_i^2 + \epsilon}} \quad \textbf{(1)}$$

where the scalars μ_i, σ_i^2 and ϵ are expanded into a vector so that each operation (addition, subtraction, division) is performed component-wise. ϵ is added in the denominator to ensure numerical stability in case that σ_i^2 is zero. We used the default of 10^{-5}. Finally, the output of the layer normalization is derived analogously to batch normalization

$$LN_i(\hat{Z}) = N_i = \gamma \odot \hat{Z}_i + \beta \qquad \gamma, \beta \in \mathbb{R}^{d_{model}} \quad \textbf{(2)}$$

where γ and β are trainable parameters of the network to allow the layer to output values with mean and variance varying from zero and one.

Calculate weights

The wights are derived on level i in the following manner:

- For each genome of the training data determine the class label for the corresponding rank i. This generates an array of class counts $G \in \mathbb{N}^{C_i}$.
- Inverse each count: $G_j^{inv} = \frac{1}{G_j}$ for $j = \{1,...,C_i\}$
- Normalize each count: $w_{ij} = \frac{G_j^{inv}}{\sum_{k=1}^{C_i} G_k^{inv}}$ for $j = \{1,...,C_i\}$

Density plots of all significant species

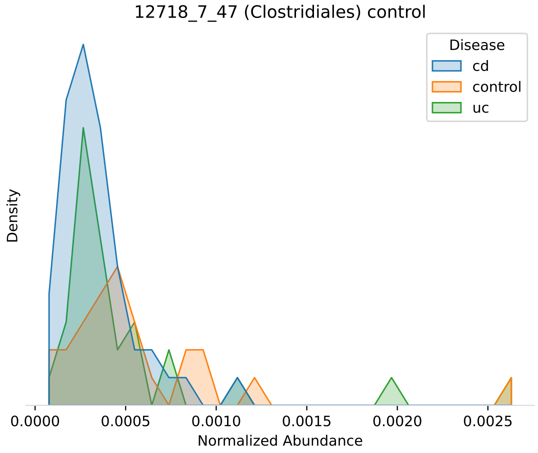

Supplementary Figure 2. Normalized abundance density of the different groups for species 12718_7_47. Crohn's disease is represented by cd, ulcerative colitis by uc and healthy subjects by control.

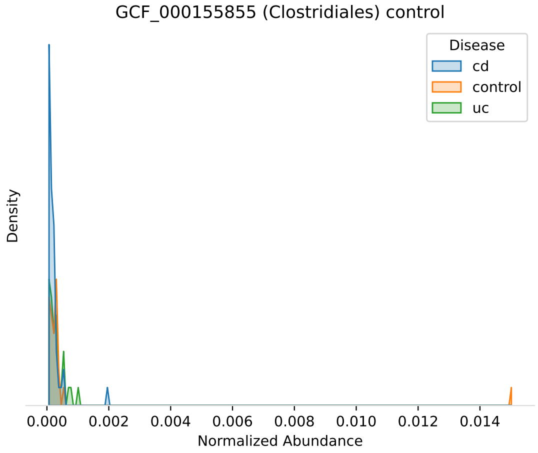

Supplementary Figure 3. Normalized abundance density of the different groups for species GCF_000155855. Crohn's disease is represented by cd, ulcerative colitis by uc and healthy subjects by control.

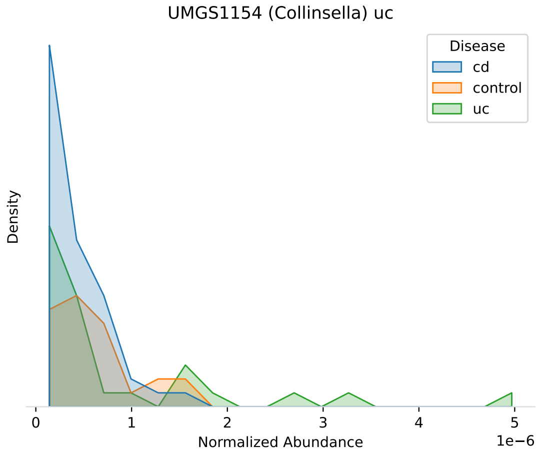

Supplementary Figure 4. Normalized abundance density of the different groups for species UMGS1154. Crohn's disease is represented by cd, ulcerative colitis by uc and healthy subjects by control.

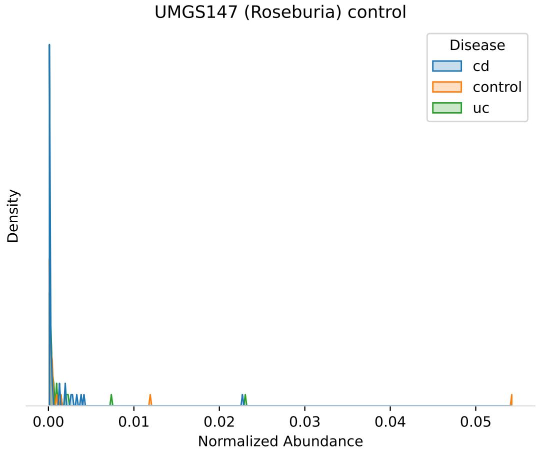

Supplementary Figure 5. Normalized abundance density of the different groups for species UMGS147. Crohn's disease is represented by cd, ulcerative colitis by uc and healthy subjects by control.

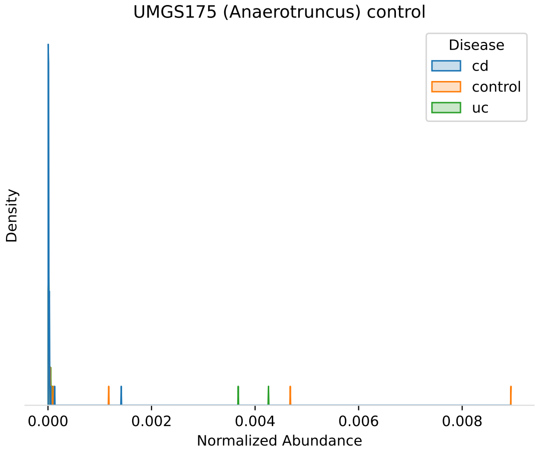

Supplementary Figure 6. Normalized abundance density of the different groups for species UMGS175. Crohn's disease is represented by cd, ulcerative colitis by uc and healthy subjects by control.

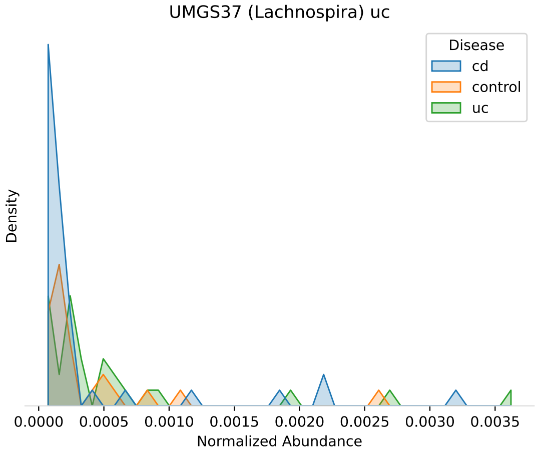

Supplementary Figure 7. Normalized abundance density of the different groups for species UMGS37. Crohn's disease is represented by cd, ulcerative colitis by uc and healthy subjects by control.

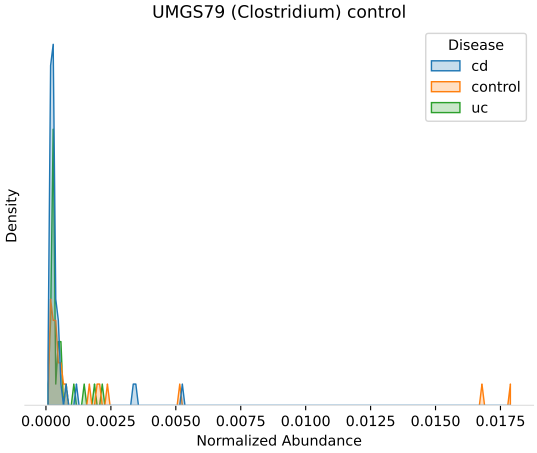

Supplementary Figure 8. Normalized abundance density of the different groups for species UMGS79. Crohn's disease is represented by cd, ulcerative colitis by uc and healthy subjects by control.

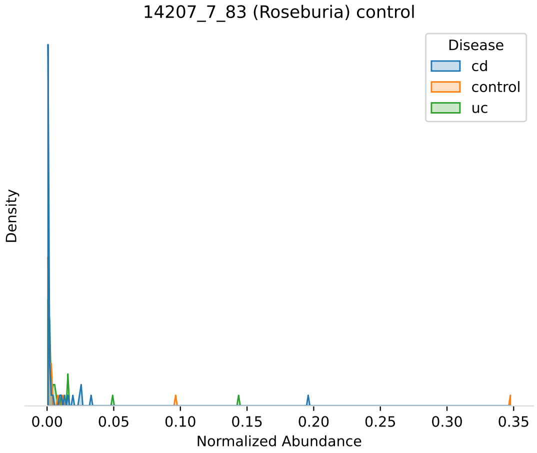

Supplementary Figure 9. Normalized abundance density of the different groups for species 14207_7_83. Crohn's disease is represented by cd, ulcerative colitis by uc and healthy subjects by control.

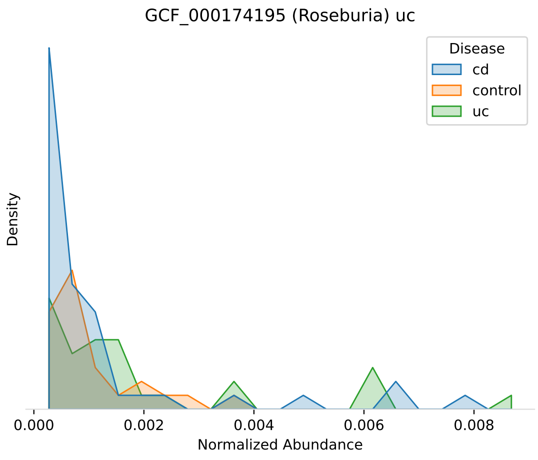

Supplementary Figure 10. Normalized abundance density of the different groups for species GCF_000174195. Crohn's disease is represented by cd, ulcerative colitis by uc and healthy subjects by control.

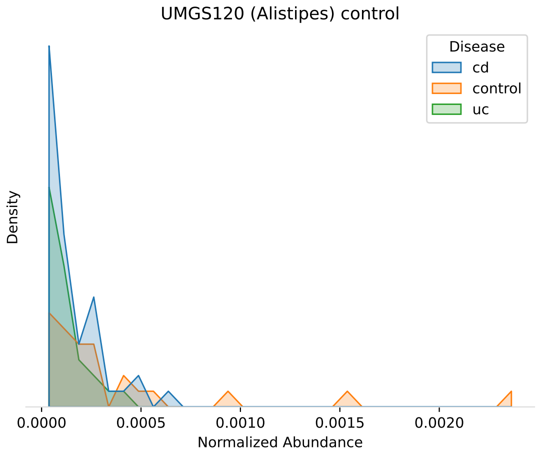

Supplementary Figure 11. Normalized abundance density of the different groups for species UMGS120. Crohn's disease is represented by cd, ulcerative colitis by uc and healthy subjects by control.

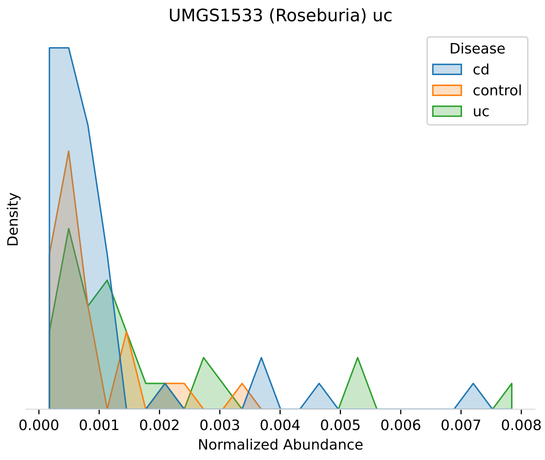

Supplementary Figure 12. Normalized abundance density of the different groups for species UMGS1533. Crohn's disease is represented by cd, ulcerative colitis by uc and healthy subjects by control.

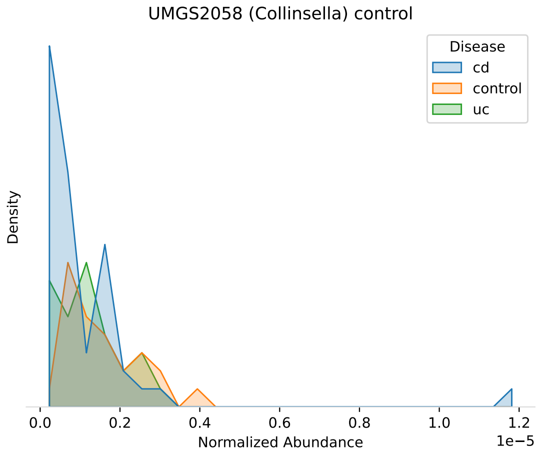

Supplementary Figure 13. Normalized abundance density of the different groups for species UMGS2058. Crohn's disease is represented by cd, ulcerative colitis by uc and healthy subjects by control.

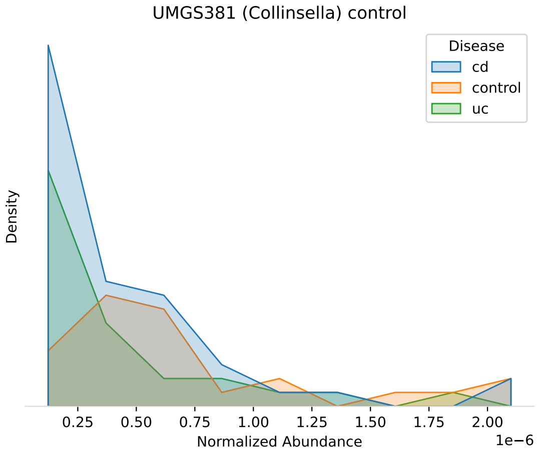

Supplementary Figure 14. Normalized abundance density of the different groups for species UMGS381. Crohn's disease is represented by cd, ulcerative colitis by uc and healthy subjects by control.

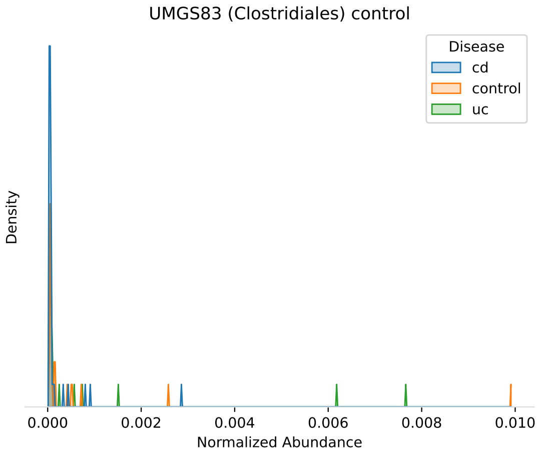

Supplementary Figure 15. Normalized abundance density of the different groups for species UMGS83. Crohn's disease is represented by cd, ulcerative colitis by uc and healthy subjects by control.

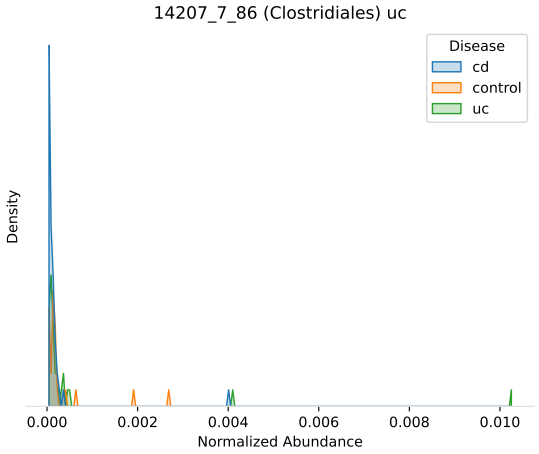

Supplementary Figure 16. Normalized abundance density of the different groups for species 14207_7_86. Crohn's disease is represented by cd, ulcerative colitis by uc and healthy subjects by control.

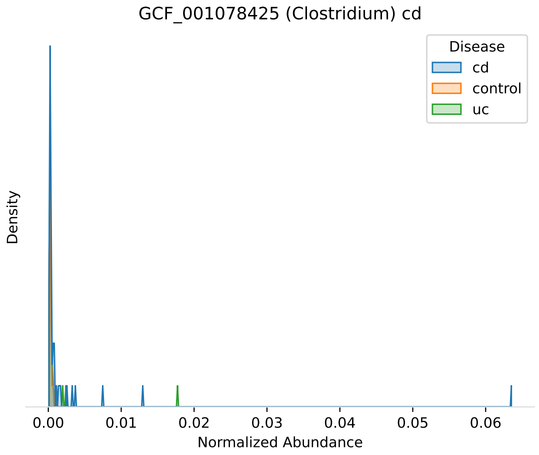

Supplementary Figure 17. Normalized abundance density of the different groups for species GCF_001078425. Crohn's disease is represented by cd, ulcerative colitis by uc and healthy subjects by control.

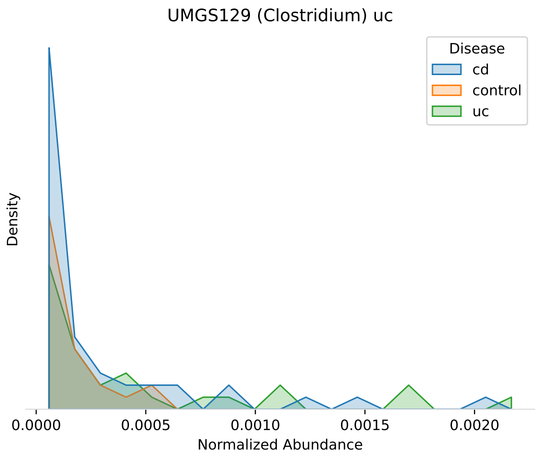

Supplementary Figure 18. Normalized abundance density of the different groups for species UMGS129. Crohn's disease is represented by cd, ulcerative colitis by uc and healthy subjects by control.

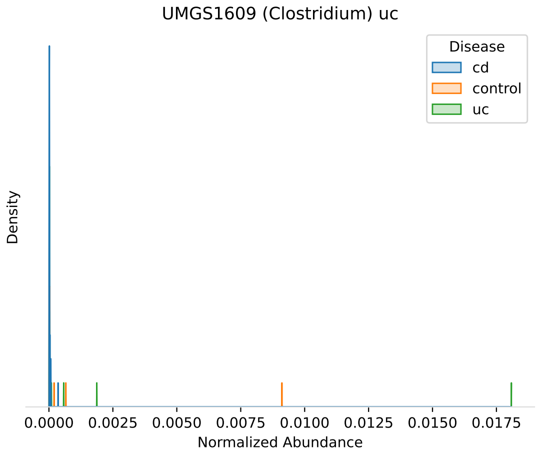

Supplementary Figure 19. Normalized abundance density of the different groups for species UMGS1609. Crohn's disease is represented by cd, ulcerative colitis by uc and healthy subjects by control.

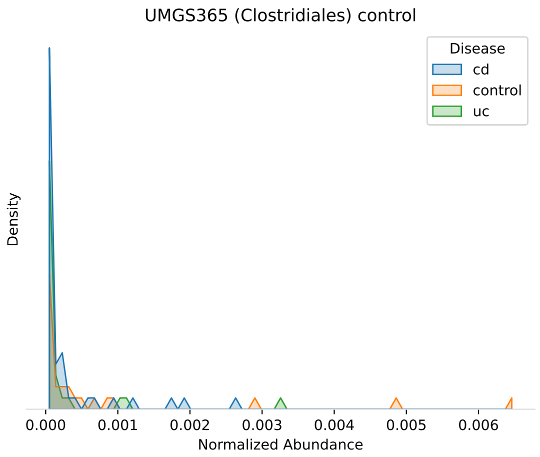

Supplementary Figure 20. Normalized abundance density of the different groups for species UMGS365. Crohn's disease is represented by cd, ulcerative colitis by uc and healthy subjects by control.

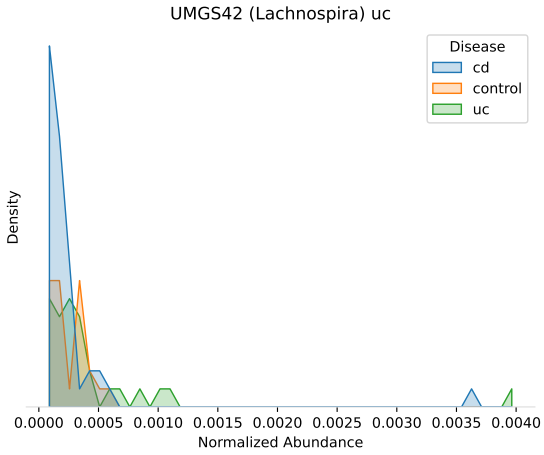

Supplementary Figure 21. Normalized abundance density of the different groups for species UMGS42. Crohn's disease is represented by cd, ulcerative colitis by uc and healthy subjects by control.

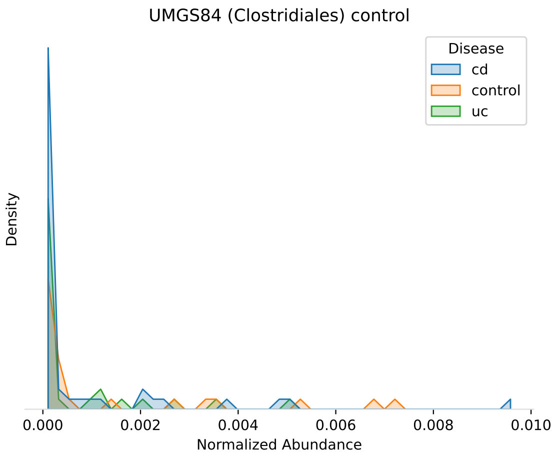

Supplementary Figure 22. Normalized abundance density of the different groups for species UMGS84. Crohn’s disease is represented by cd, ulcerative colitis by uc and healthy subjects by control.

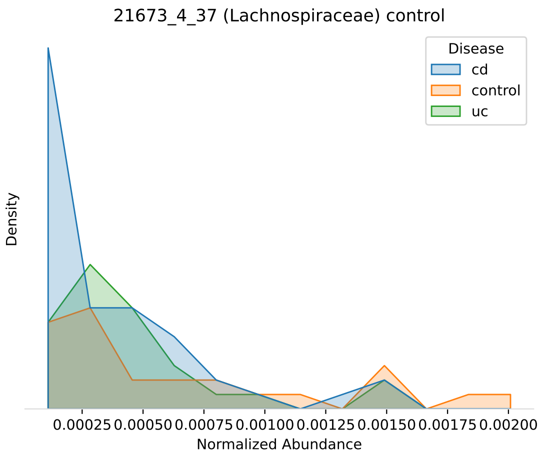

Supplementary Figure 23. Normalized abundance density of the different groups for species 21673_4_37. Crohn’s disease is represented by cd, ulcerative colitis by uc and healthy subjects by control.

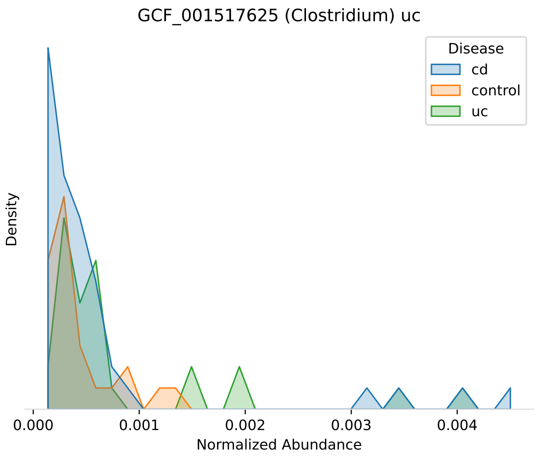

Supplementary Figure 24. Normalized abundance density of the different groups for species GCF_001517625. Crohn’s disease is represented by cd, ulcerative colitis by uc and healthy subjects by control.

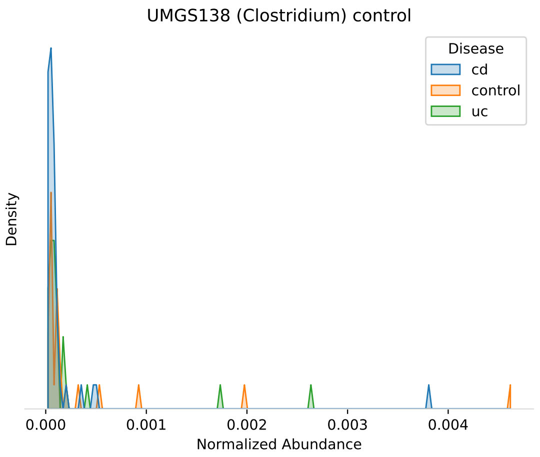

Supplementary Figure 25. Normalized abundance density of the different groups for species UMGS138. Crohn’s disease is represented by cd, ulcerative colitis by uc and healthy subjects by control.

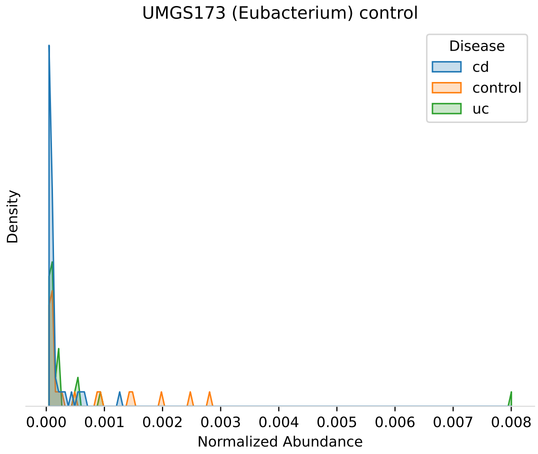

Supplementary Figure 26. Normalized abundance density of the different groups for species UMGS173. Crohn’s disease is represented by cd, ulcerative colitis by uc and healthy subjects by control.

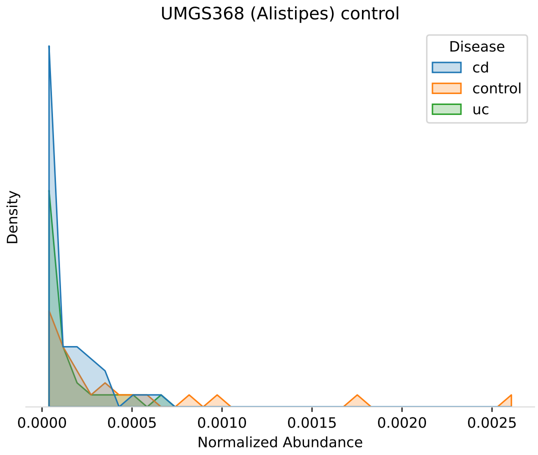

Supplementary Figure 27. Normalized abundance density of the different groups for species UMGS368. Crohn’s disease is represented by cd, ulcerative colitis by uc and healthy subjects by control.

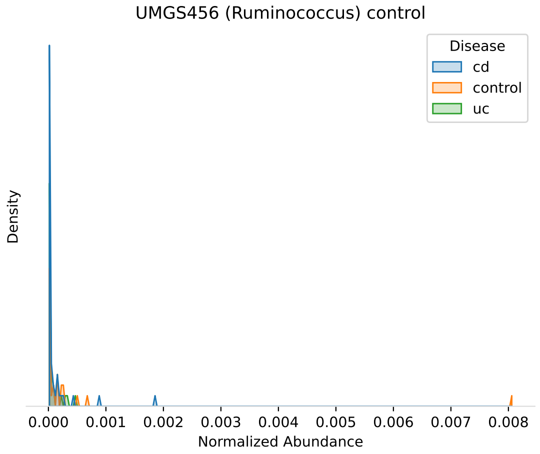

Supplementary Figure 28. Normalized abundance density of the different groups for species UMGS456. Crohn’s disease is represented by cd, ulcerative colitis by uc and healthy subjects by control.

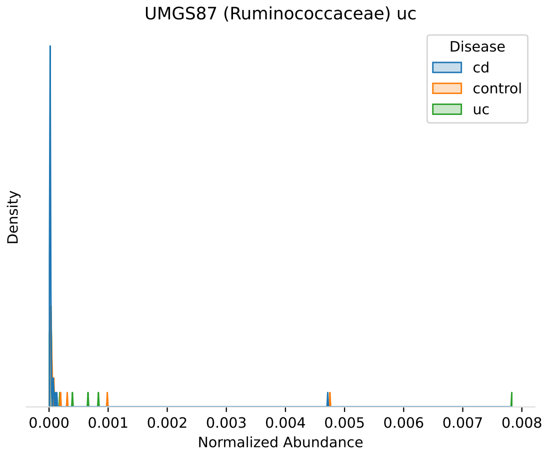

Supplementary Figure 29. Normalized abundance density of the different groups for species UMGS87. Crohn’s disease is represented by cd, ulcerative colitis by uc and healthy subjects by control.

REFERENCES

1. Georgiou, A., Fortuin, V., Mustafa, H., and Rätsch, G. (2019) META^2: Memory-efficient taxonomic classification and abundance estimation for metagenomics with deep learning. *arXiv,* doi: https://doi.org/10.48550/arXiv.1909.13146, 11 January 2023, pre-print: not peer-reviewed.
2. Leskovec, J., Rajaraman, A., and Ullman, J. D. (2014) Mining of Massive Datasets, Cambridge University Press, USA 2nd edition.
3. Broder, A. (June, 1997) On the resemblance and containment of documents. In *Proceedings. Compression and Complexity of SEQUENCES)* pp. 21–29.
4. Wolf, T., Debut, L., Sanh, V., Chaumond, J., Delangue, C., et al. (2019) HuggingFace's Transformers: State-of-the-art Natural Language Processing. *arXiv,* doi: https://doi.org/10.48550/arXiv.1910.03771, 11 January 2023, pre-print: not peer-reviewed.
5. Nguyen, H. V., Patel, V. M., Nasrabadi, N. M., and Chellappa, R. (2012) Sparse Embedding: A Framework for Sparsity Promoting Dimensionality Reduction. In *ECCV* Berlin, Heidelberg: Lecture Notes in Computer Science pp. 414–427.
